# Two novel colorimetric fluorescent probes: Hg^2+^ and Al^3+^ in the visual colorimetric recognition environment†

**DOI:** 10.1039/c9ra08428b

**Published:** 2020-01-16

**Authors:** Wenxia Zhong, Lizhen Wang, Shimin Fang, Dawei Qin, Jianhua Zhou, Geng Yang, Hongdong Duan

**Affiliations:** School of Chemistry and Pharmaceutical Engineering, Qilu University of Technology (Shandong Academy of Sciences) Ji'nan Shandong Province China 250353 hdduan67@163.com +86 13153035598; Biology Institute, Qilu University of Technology (Shandong Academy of Sciences) Ji'nan Shandong Province China 250014

## Abstract

Two new dual channel Schiff base fluorescent probes, Tri-R6G and Tri-Flu, were synthesized, and can detect Hg^2+^ and Al^3+^, respectively. The two probes were characterized by FTIR, ^1^H NMR, ^13^C NMR and HRMS, and their optical properties were detected by UV and FL. Test results showed the probes' detection of Hg^2+^ and Al^3+^ compared to other metal ions (Ag^+^, Co^2+^, Cd^2+^, Mg^2+^, Cu^2+^, Ni^2+^, Ba^2+^, Pb^2+^, Cr^3+^, Al^3+^, Zn^2+^, Hg^2+^, K^+^, Ga^2+^ and Fe^3+^), respectively. Besides, the detection limits were determined to be 1.61 × 10^−8^ M and 1.15 × 10^−8^ M through the standard curve plot, respectively. The photoelectron transfer (PET) mechanism was guessed by the Job's plot and the infrared titration. Corresponding orbital electron distribution and molecular geometry configurations of the compounds were predicted by density functional theory (DFT). In addition, the prepared test paper changed from white to pink when the target ion was detected. The color changed from colorless to pink in a solution having a concentration of 10^−5^ M.

## Abstract 2

Two new dual channel Schiff base fluorescent probes, Tri-R6G and Tri-Flu, were synthesized, and can detect Hg^2+^ and Al^3+^, respectively.

## Graphical abstract

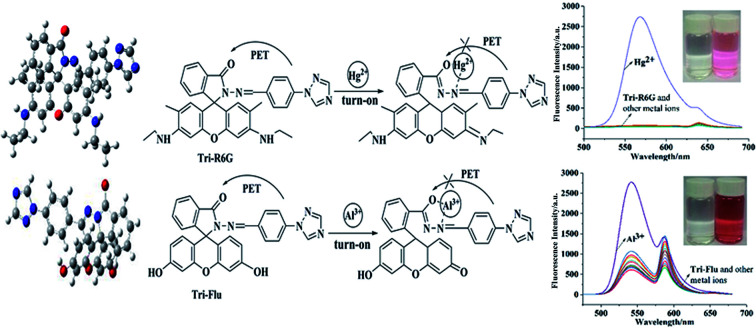

## 1. Introduction

With the rapid development of industries, metal ion pollution has become a serious environmental problem.^1–6^ Mercury is the only metal that exists as a liquid state, and at room temperature, mercury can evaporate into the highly toxic mercury vapor.^7–13^ Even worse is that mercury can be enriched by passive plants and converted into more toxic organic mercury through biotransformation, which can easily enter the human body and cause various toxic effects in the human body. For example, prolonged exposure to high concentrations of mercury usually causes brain damage and death.^14–16^ The latest statistics revealed that the amount of mercury ingested by a human had reached 20–30 μg d^−1^, and in heavily polluted areas, it was as high as 200–300 μg d^−1^ which poses serious threats to human health.^17,18^ Therefore, the prevention and control of mercury pollution has become an urgent problem to be solved for countries all over the world. On the other hand, aluminum is an amphoteric element that reacts with both acids and alkalis.^19–27^ Compounds formed after the reaction are easily absorbed by the intestines and can enter the brain, thus affecting the mental development of children and leading to senile dementia. Studies have shown that aluminum can cause decreased exercise and learning and memory, and affect children's mental development. Aluminum can also affect the reproductive capacity of male animals and inhibit fetal growth and development, and cause bone problems through interaction with calcium and phosphorus systematic damage and deformation, rickets, osteoporosis, *etc.* Consequently, the detection of aluminum is particularly important.^28–30^

Schiff base fluorescent probes have been widely used for detection of metal ions owing to their high sensitivity, low detection concentrations and perfect stability.^31–37^ The C=N bond in Schiff fluorescent probes can form a stable complex with metal ions accompanied with an obvious fluorescence intensity enhancement or attenuation, which can be applied for detecting metal ions. Therefore, many Schiff base fluorescent probes have been developed for highly selective and sensitive detecting Hg^2+^ and Al^3+^.^38–44^ For example, the Mahajan group^45^ reported a novel pyridazine-oxime-based fluorescent probe that enhanced fluorescence recognition of Hg^2+^ at 550 nm in DMSO/H_2_O (8/2, v/v) solution with a low detection limit (26.1 nM). This probe was further applied to detect Hg^2+^ in human cervical cancer HeLa cells, which indicated its potential applicable in biological samples. The Hou group^46^ synthesized a turn-on fluorescent probe that could be complexed with Al^3+^ in a ratio of 1 : 1 to change the color of the solution from pale yellow to yellow. The detection limit of this probe was 82.2 nM and the fluorescence was significantly enhanced at 480 nm, which had potential application value in sensor development. However, the above-mentioned fluorescent probes usually need tedious synthesis routines and are insensitive for detecting low concentration metal ions. Therefore, it is necessary to develop highly sensitive and selective fluorescent probes for detection of Hg^2+^ and Al^3+^.

**Scheme 1:**
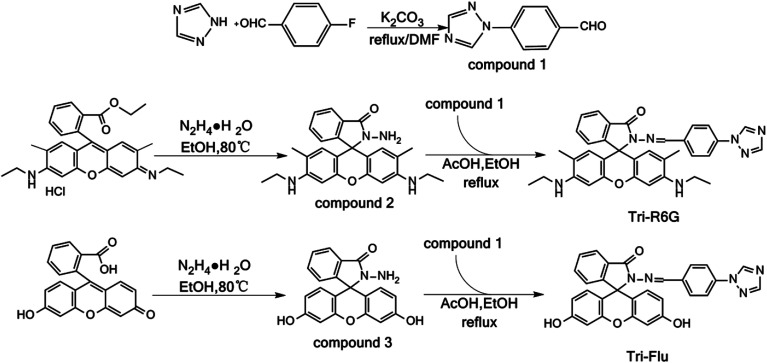
Synthesis of probes Tri-R6G and Tri-Flu.

Triazole, a nitrogen-containing heterocyclic compound having unique biological activities, low toxicity and high systemicity,^47–51^ has been widely used as key intermediates in the synthesis of various pesticides or pharmaceutical. Neupane group^52^ reported a triazolyl complex based on a photoelectron transfer (PET) mechanism, which could detect Hg^2+^ under neutral and weakly acidic conditions with the detection limit of 672 nM. Chereddy group^53^ synthesized a rhodamine-based fluorescent probe containing a 1,2,3-triazole ring under the guidance of soft and hard acid–base theory. It had 1 : 1 binding with Cu^2+^ and the detection limit was 60 nM, which could be used to detect Cu^2+^ biological samples. Two novel Schiff base fluorescent probes are synthesized, by grafting triazole on Rhodamine 6G and fluorescein derivatives, triazolyl benzaldehyde, Rhodamine 6G internal hydrazide Schiff base and triazolyl benzaldehyde fluorescein acyl hydrazide Schiff base (Tri-R6G and Tri-Flu).The structures of Tri-R6G and Tri-Flu were confirmed by FTIR, ^1^H NMR, ^13^C NMR and HRMS, and they were detected Hg^2+^ and Al^3+^ by UV and FL. The sensitive detection limits of these two probes were 1.61 × 10^−8^ M and 1.15 × 10^−8^ M, respectively. In addition, we used the probe to prepare the test paper, the prepared test paper changed from white to pink when the target ion was detected. The PET mechanism was guessed by Job's plot and infrared titration plot and was supported by theoretical calculations (Scheme 1).

## 2. Experimental section

### 2.1 General procedure

The solvents and drugs used in the experiments were all analytical grade and obtained from commercial sources. (1,2,4-Triazole, Rhodamine 6G and Fluorescein were purchased from Aladdin). The ^1^H NMR (400 MHz) and ^13^C NMR (100 MHz) spectra were measured on a Bruker AV-400 spectrometer with DMSO-*d*_6_ used as the solvent and tetramethylsilane (TMS) as the internal standard. Infrared measurements were performed using a KBr pellet technique on a Bruker ALPHA FT-IR spectrometer in the 4000–400 cm^−1^ region. High resolution mass spectrometry (HRMS) was performed using an Agilent 6510 precision mass Q-TOF LC/MS system. Fluorescence spectra were recorded on a Hitachi Fl-4600 fluorescence spectrophotometer with a scan rate of 2400 nm min^−1^. Ultraviolet-visible (UV-vis) absorption were measured on SHIMADZU UV-2600.All experiments were performed at room temperature.

### 2.2 Synthesis

#### 2.2.1 Synthesis of compound 1^31^

All glassware were cleaned with freshly lye, subsequently rinsed with copious amount of distilled water, and dried well before use. In a typical experiment, in a 250 mL three-necked, round-bottomed flask, was added 1.40 g of 1,2,4-triazole (20.19 mmol), 2.48 g of *p*-fluorobenzaldehyde (19.96 mmol), and 3.09 g of anhydrous potassium carbonate (22.39 mmol) dissolved in 100 mL of DMF, the reaction was allowed to proceed under vigorous stirring at 100 °C. After 24 hours, the reaction was cooled to 25 °C and poured into 300 mL of ice-water. Some buff solids precipitated, which was subjected to filtration, and the filtrate was recrystallized with ethyl acetate and dried in vacuum to give compound 1 as a white solid (2.69 g, yield 91%).

##### Triazole benzaldehyde (compound 1)

IR (KBr): 1689, 1604, 1519, 1207, 981, 837 cm^−1^; ^1^H NMR (DMSO-*d*_6_, 400 MHz): *δ* 10.02 (s, 1H), 8.45 (s, 1H), 8.04 (d, *J* = 8.5 Hz, 2H), 7.92 (d, *J* = 8.8 Hz, 2H), 7.16 (s, 1H); ^13^C NMR (DMSO-*d*_6_, 100 MHz): *δ* 165.91, 158.65, 152.86, 133.04, 129.80, 128.85, 123.87, 122.80, 112.44, 110.40, 102.81, 65.07, 56.48, 40.61, 40.19; ESI-TOF HRMS (*m*/*z*): calcd for C_9_H_7_N_3_O, [M + H]^+^, 174.1405; found, 173.1715.

#### 2.2.2 Synthesis of compound 2

Rhodamine 6G (5.06 g, 10.56 mmol) and hydrazine (1.78 g, 35 mmol) were placed in a flask, and anhydrous ethanol was added as a solvent. The reaction mixture was allowed to warm 78 °C under a refluxing device and stirred at ambient temperature for 4 hours, a solid precipitated was formed. The mixture was filtered and the filtrate was washed with hot ethanol and dried under vacuum to give compound 2 as a colorless solid (4.85 g, yield 79%).

##### Rhodamine 6G hydrazide (compound 2)

IR (KBr): 3417, 3101, 2966, 1714, 1622, 1519, 1467, 1276 cm^−1^; ^1^H NMR (DMSO-*d*_6_, 400 MHz): *δ* 7.77–7.73 (m, 1H), 7.49–7.42 (m, 2H), 6.95–6.90 (m, 1H), 6.26 (s, 2H), 6.10 (s, 2H), 4.99 (t, *J* = 5.3 Hz, 2H), 4.21 (s, 2H), 3.13 (p, *J* = 6.8 Hz, 4H), 1.88 (d, *J* = 9.9 Hz, 6H), 1.21 (t, *J* = 7.0 Hz, 6H); ^13^C NMR (DMSO-*d*_6_, 100 MHz): *δ* 165.18, 152.05, 151.31, 147.34, 132.23, 129.45, 127.97, 126.96, 123.40, 122.11, 117.77, 104.95, 95.86, 64.99, 37.44, 17.03, 14.13; ESI-TOF HRMS (*m*/*z*): calcd for C_26_H_28_N_4_O_2_, [M + H]^+^, 429.2375; found, 428.2212.

#### 2.2.3 Synthesis of compound 3^54^

Fluorescein (4.89 g, 14.69 mmol) and hydrazine (1.24 g, 24.77 mmol) were placed in a flask, and anhydrous ethanol was added as a solvent. The reaction mixture was heated to 78 °C and stirred at ambient temperature for 5 hours. The reaction was cooled to 25 °C and poured into 300 mL of ice water to give a white solid. The mixture was filtered and the filtrate was washed with ice-water and dried under vacuum to give compound 3 as a yellow solid (4.03 g, yield 66%).

##### Fluorescein endonyl hydrazide (compound 3)

IR (KBr): 3439, 3121, 2805, 1713, 1689, 1609, 1587, 1217 cm^−1^; ^1^H NMR (DMSO-*d*_6_, 400 MHz): *δ* 9.62 (s, 2H), 7.79 (m, 1H), 7.45–7.52 (m, 2H), 6.97 (m, 1H), 6.58–6.59 (d, *J* = 2.4 Hz, 2H), 6.38–6.47 (m, 4H), 4.37 (s, 2H); ^13^C NMR (DMSO-*d*_6_, 100 MHz): *δ* 192.54, 153.47, 143.59, 141.19, 135.45, 131.74, 119.95, 56.47, 19.01; ESI-TOF HRMS (*m*/*z*): calcd for C_20_H_14_N_2_O_4_, [M + H]^+^, 347.1161; found, 346.0954.

#### 2.2.4 Synthesis of Tri-R6G

In a 250 mL three-necked, round-bottomed flask, was added 0.87 g of compound 1 (50.28 mmol), 2.23 g of compound 2 (52.10 mmol) and glacial acetic acid dissolved in 100 mL of DMF, the reaction was allowed to proceed under vigorous stirring at 100 °C. After 5 hours, 200 mL of ice water was added and some pink solids precipitated. The solid was resuspended with acetone, crystallized and dried under vacuum to give Tri-R6G as a pink solid (2.05 g, yield 66%).

##### Tri-R6G

IR (KBr): 3421, 2976, 1689, 1611, 1502, 1368, 1146 cm^−1^; ^1^H NMR (DMSO-*d*_6_, 400 MHz): *δ* 9.30 (s, 1H), 8.76 (s, 1H), 8.24 (s, 1H), 7.92 (d, *J* = 7.2 Hz, 1H), 7.86 (d, *J* = 8.6 Hz, 2H), 7.64–7.54 (m, 4H), 7.06 (d, *J* = 7.3 Hz, 1H), 6.34 (s, 2H), 6.18 (s, 2H), 5.07 (t, *J* = 5.3 Hz, 2H), 3.18–3.09 (m, 4H), 1.85 (d, *J* = 9.1 Hz, 6H), 1.20 (t, *J* = 7.1 Hz, 6H); ^13^C NMR (DMSO-*d*_6_, 100 MHz): *δ* 163.76, 152.52, 151.06, 147.80, 142.38, 137.54, 133.92, 128.74, 127.95, 126.84, 123.81, 123.02, 119.55, 118.22, 105.06, 95.75, 37.43, 16.94, 14.11; ESI-TOF HRMS (*m*/*z*): calcd for C_35_H_33_N_7_O_2_, [M + H]^+^, 584.2752; found, 583.2696.

#### 2.2.5 Synthesis of Tri-Flu

In a 250 mL three-necked, round-bottomed flask, was added 0.18 g of compound 1 (1.04 mmol), 0.52 g of compound 3 (1.21 mmol) and glacial acetic acid dissolved in 100 mL of ethanol, the reaction was allowed to proceed under vigorous stirring at 78 °C. After 5 hours, 200 mL of ice water was added and some buff solids precipitated. The solid was resuspended with acetone, crystallized and dried under vacuum to give Tri-Flu as a buff solid (0.44 g, yield 63%).

##### Tri-Flu

IR (KBr): 3427, 3120, 1697, 1613, 1509, 1450, 1315 cm^−1^; ^1^H NMR (DMSO-*d*_6_, 400 MHz): *δ* 9.93 (s, 2H), 9.31 (s, 1H), 9.13 (s, 1H), 8.25 (s, 1H), 7.94 (d, *J* = 7.3 Hz, 1H), 7.87 (d, *J* = 8.5 Hz, 2H), 7.62 (dd, *J* = 14.4, 6.6 Hz, 4H), 7.16 (d, *J* = 7.4 Hz, 1H), 6.67 (d, *J* = 2.0 Hz, 2H), 6.49 (dd, *J* = 18.9, 9.6 Hz, 4H); ^13^C NMR (DMSO-*d*_6_, 100 MHz): *δ* 163.63, 158.57, 152.31, 150.18, 128.02, 119.54, 112.30, 110.21, 102.45, 55.98, 18.51; ESI-TOF HRMS (*m*/*z*): calcd for C_29_H_19_N_5_O_4_, [M + H]^+^, 502.1501; found, 501.1437.

## 3. Results and discussion

### 3.1 UV-vis absorption spectra

**Fig. 1:**
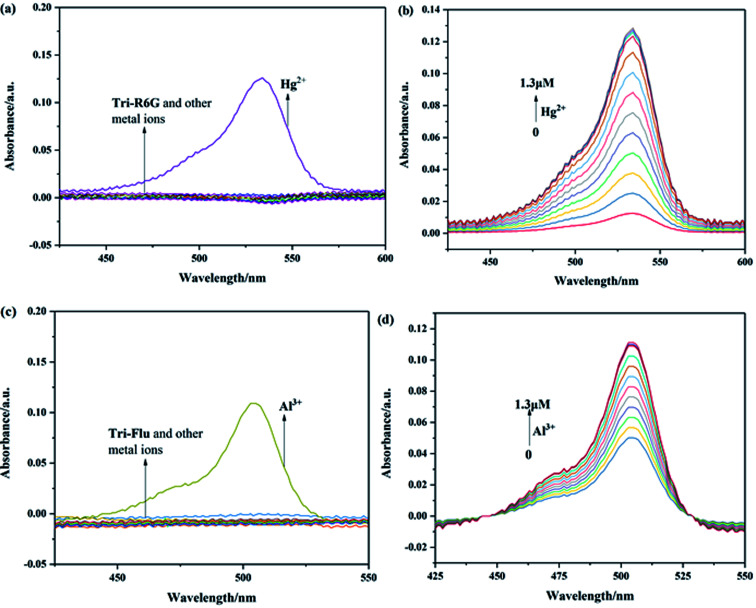
UV-visible absorption of Tri-R6G (a) and Tri-Flu (c) in the presence and absence of different metal ions in a mixed solution of DMSO/H_2_O (7/3, v/v) at 25 °C. (b) and (d) Upon titration with Hg^2+^ and Al^3+^ (1 × 10^−5^ M, 0–1.3 μM), respectively.

The recognition abilities of probes Tri-R6G and Tri-Flu were investigated in a mixed solution of DMSO/H_2_O (7/3, v/v, 10 μM) in the presence and absence of different metal ions including Ag^+^, Co^2+^, Cd^2+^, Mg^2+^, Cu^2+^, Ni^2+^, Ba^2+^, Pb^2+^, Cr^3+^, Al^3+^, Zn^2+^, Hg^2+^, K^+^, Ga^2+^ and Fe^3+^. As shown in Fig. 1(a), the absorption spectrum of Tri-R6G in the solution of DMSO/H_2_O (7/3, v/v, 10 μM) exhibited a low energy band centered at 534 nm. The addition of Hg^2+^ resulted in an obvious color change from colorless to pink, whereas adding other metal ions could not lead to any color changes. In Fig. 1(b), the intensity of the absorption band at 534 nm increased distinctly upon gradual addition of Hg^2+^ (0–1.3 μM). The transparent solution of Tri-R6G changed to pink instantly after addition of Hg^2+^ ion. These results proved probe Tri-R6G exhibited high selectivity toward Hg^2+^ on the UV-vis spectrum. The absorption band appeared at 534 nm might be caused by the ring opening of the lactam. Similarly, the UV-vis absorption of probe Tri-Flu also showed two bands as shown in Fig. 1(c), a weak energy band at 504 nm. In Fig. 1(d), the intensity of the absorption band at 504 nm increased distinctly upon gradual addition of Al^3+^ (0–1.3 equiv.). The transparent solution of Tri-Flu changed to pink instantly after addition of Al^3+^ ion. Compared with other metal ions, the addition of Al^3+^ resulted in an obvious color change from colorless to pink, which proved the high selectivity of probe Tri-Flu toward Al^3+^.

### 3.2 Fluorescence spectra

**Fig. 2:**
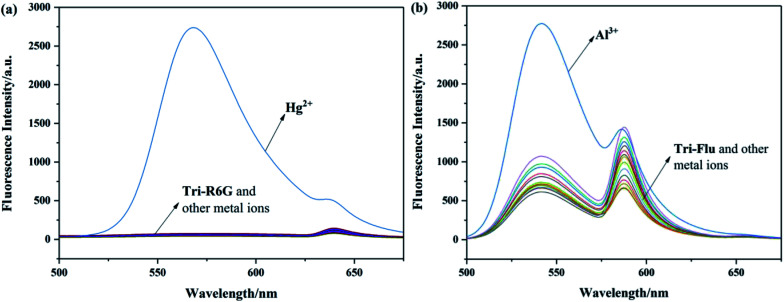
The fluorescence spectra of Tri-R6G (a) (10 μM) and Tri-Flu (b) (10 μM) in the presence and absence of different metal ions (10 μM) in DMSO/H_2_O (7/3, v/v), respectively.

**Fig. 3:**
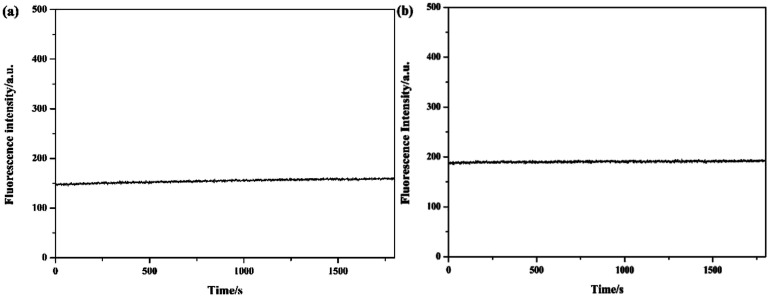
The photo stability experiments of probes Tri-R6G (a) and Tri-Flu (b).

To investigate the optical properties of probes Tri-R6G and Tri-Flu, their fluorescence spectra toward various metal ions were explored in a mix solution of DMSO/H_2_O (10 μM) as shown in Fig. 2. Free sensor Tri-R6G showed a weak absorption band at 560 nm, whereas that of sensor Tri-Flu was at 541 nm. After addition of Hg^2+^, the solution of probe Tri-R6G showed an obvious and strong absorption band at 560 nm (Fig. 2(a)), while addition of other metal ions elicited no or weak fluorescence enhancement that could be ignored. These results revealed again the high selectivity and specificity of probe Tri-R6G against Hg^2+^. In addition, with Rhodamine 6G as the reference material, the relative fluorescence quantum yield of probe Tri-R6G is 0.1089 (*Y*_f_ = *k*_f_/(*k*_f_ + Σ*k*_i_)).The similar results were observed in the fluorescence spectra of Tri-Flu (Fig. 2(b)), which exhibited high selectivity toward Al^3+^ compared with various other metal ions added to the solution. When probes Tri-R6G and Tri-Flu bind to metal ions Hg^2+^ and Al^3+^, their fluorescence intensity is steady, and no obvious attenuation within half an hour (Fig. 3(a) and (b)). Therefore, probe Tri-R6G and Tri-Flu can be used to detect Hg^2+^ and Al^3+^, respectively.

**Fig. 4:**
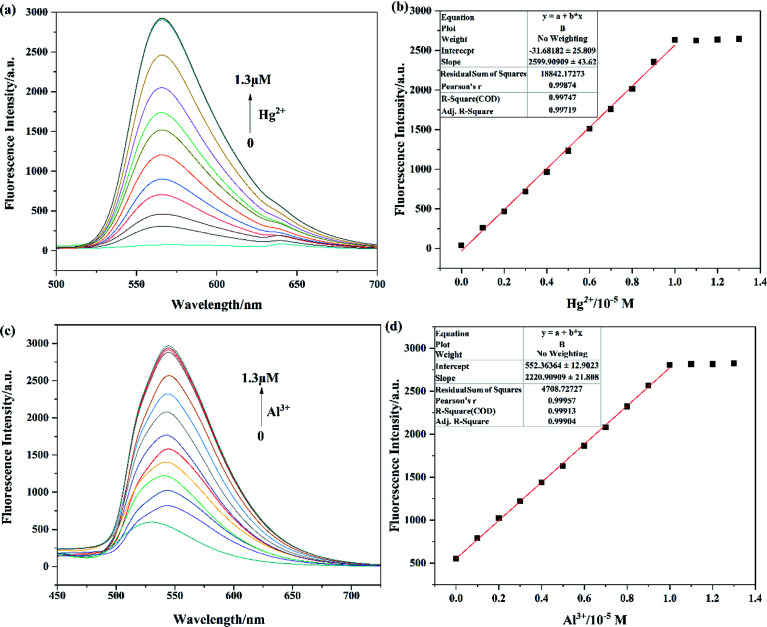
The fluorescence spectra of Tri-R6G (a) and Tri-Flu (c) (10 μM) with the increasing concentration of Hg^2+^ and Al^3+^ ions (0–1.3 μM) in DMSO/H_2_O (7/3, v/v) and ethanol, respectively; the linear fit between Tri-R6G (b) with Hg^2+^ ions and Tri-Flu (d) with Al^3+^ ions.

The sensitivities of probes Tri-R6G toward Hg^2+^ and Tri-Flu toward Al^3+^ were investigated through fluorescence titration experiments as described in Fig. 4. With the concentration of Hg^2+^ added to the solution increasing, fluorescence spectra of probe Tri-R6G showed significant enhancement (Fig. 4(a)), which could be ascribed to the coordination reaction of Tri-R6G with Hg^2+^ and consequently decreased the PET process and C=N isomerization. In addition, there is a good linear relationship (*R*_1_ = 0.99874) between fluorescence intensity and Hg^2+^ concentration (Fig. 4(b)). The fluorescence titration experiment of Tri-Flu toward Al^3+^ was also carried out as shown in Fig. 4(c). With the increase of Al^3+^ concentration, the fluorescence intensity of Tri-Flu increased significantly and showed a good linear relationship (Fig. 4(d), *R*_2_ = 0.99957). Furthermore, according to the formula DL = 3*σ*/*k*, the detection limit of Tri-R6G toward Hg^2+^ was calculated to be 1.61 × 10^−8^ M, and that of Tri-Flu toward Al^3+^ was 1.15 × 10^−8^ M. These results demonstrated the high sensitivity of the synthetic probes and their potential application for the detection of Hg^2+^ and Al^3+^.

**Fig. 5:**
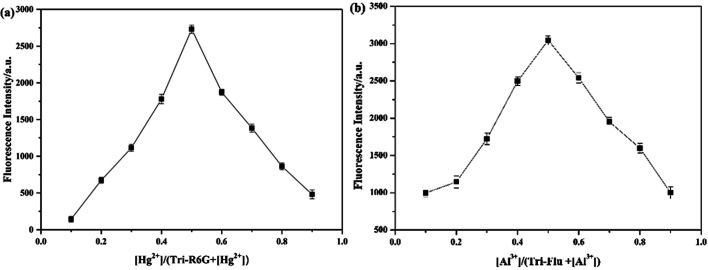
Job's plot for determining the stoichiometry of Tri-R6G + Hg^2+^ (a) in DMSO/H_2_O (7/3, v/v) solution and Tri-Flu + Al^3+^ (b) in ethanol solution.

When more than 1 equiv. of Hg^2+^ was added, no change in the fluorescence intensity was observed. This result might prove that the binding stoichiometry of Tri-R6G to Hg^2+^ was 1 : 1. Therefore, a drawing experiment of Job was performed to study the combined stoichiometry between the probes and the metal ions. As shown in Fig. 5(a), the maximum fluorescence intensity of a mole fraction of about 10^−5^ M was measured, and it is shown that a complex is formed between Tri-R6G and Hg^2+^ in a mole ratio of 1 : 1. Similarly, the formation of a complex between Tri-Flu and Al^3+^ in a ratio of 1 : 1 can be observed in Fig. 5(b).

### 3.3 Competition experiments

**Fig. 6:**
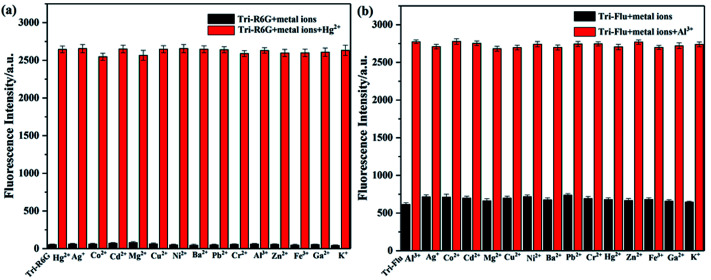
The black bars represent the fluorescent intensity of sensor Tri-R6G (a) or Tri-Flu (b) and various metal ions (1.0 equiv.) in solution; the red bars represent the fluorescent intensity of sensor Tri-R6G + Hg^2+^ (a) or Tri-Flu + Al^3+^ (b) (1.0 equiv.) in solution.

High selectivity is an important parameter for studying the performance of fluorescent probes. Therefore, the competition experiment was carried out to examine the binding ability of synthetic probes toward metal ions in a mix solution of DMSO and H_2_O, and the metal concentrations used in this experiment were all 10^−5^ M. As shown in Fig. 6(a), addition of Hg^2+^ to a solution of Tri-R6G leaded to a mixture with obvious fluorescence enhancement, which could not affect by adding other metal ions to the mixture, indicating the high selectivity of probe Tri-R6G. Similar experiments depicted Fig. 6(b) also proved the high selectivity of probe Tri-Flu toward Al^3+^.

### 3.4 pH effects

**Fig. 7:**
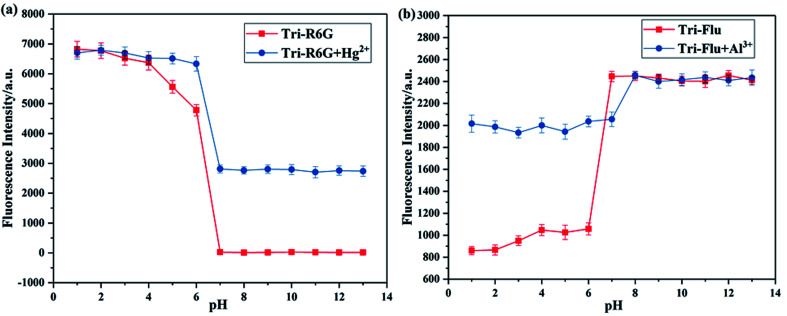
Fluorescence response of sensor Tri-R6G (a) and Tri-Flu (b) (10 μM) as a function of pH (pH 1.0–13.0) in the absence and presence of Hg^2+^ and Al^3+^ (10 μM), receptively, at room temperature.

It has been well proved that pH value is essential for the detection ability of fluorescent probes. Hence, the effect of pH on fluorescence intensity was investigated to evaluate the potential applicability of probes Tri-R6G and Tri-Flu as shown in Fig. 7. Clearly, the fluorescence intensity of probe Tri-R6G significantly reduced with the increase of pH when it was below 6, whereas the fluorescence intensity was observed to be held steady between pH 7–13, when Hg^2+^ was added, the probe also had strong fluorescence intensity at pH 7 or higher. Which proved that probe Tri-R6G could test Hg^2+^ within a biological scale of pH values. Oppositely, the fluorescence intensity of probe Tri-Flu significantly increased with the increase of pH when it was above 6, but at high pH value (>7), the fluorescence intensity tends to saturation. When Al^3+^ was added, the probe also had strong fluorescence intensity when the pH is below 7. Which indicated that Tri-Flu can be used in acidic environments.

### 3.5 Colorimetric experiment

**Fig. 8:**
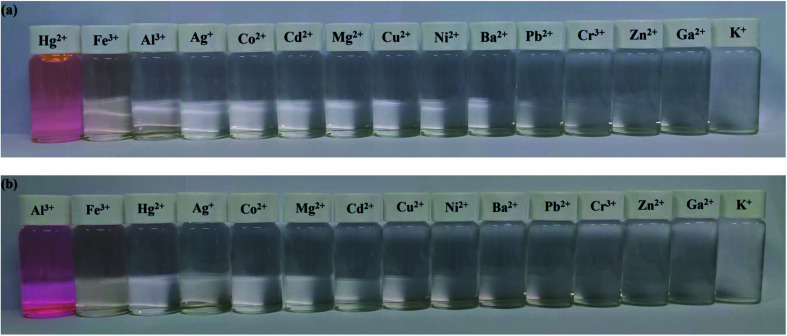
Colorimetric performance of sensor Tri-Flu + Hg^2+^ (a) (10 μM) and Tri-R6G + Al^3+^ (b) (10 μM) upon addition of different metal ions (1.0 μM) in DMSO/H_2_O (7/3, v/v) solution and ethanol solution.

The specificity of probes Tri-R6G and Tri-Flu toward Hg^2+^ and Al^3+^ were also investigated by colorimetric experiments, respectively. As shown in Fig. 8(a), addition of the same concentration of Hg^2+^ was to a solution of sensor Tri-R6G in DMSO/H_2_O (7/3, v/v, 10 μM) caused obvious color change from colorless to pink and the whole process was finished only in one second, suggesting that sensor Tri-R6G could be used for naked-eye recognition of Hg^2+^ with concentration as low as 10 μM. While, the addition of other metal ions showed almost no color changes in absorption peak, which demonstrated the high specificity of probe Tri-R6G for detecting Hg^2+^. Similarly, an obvious color change from colorless to pink was observed after addition of the same concentration of Al^3+^ to a solution of Tri-Flu in DMSO/H_2_O (7/3, v/v, 10 μM) as shown in Fig. 8(b), the same sensor showed no obvious color changes in the presence of other metal ions. These results proved that probe Tri-Flu could be employed as a high sensitivity sensor for naked eye recognition of Al^3+^.

**Fig. 9:**
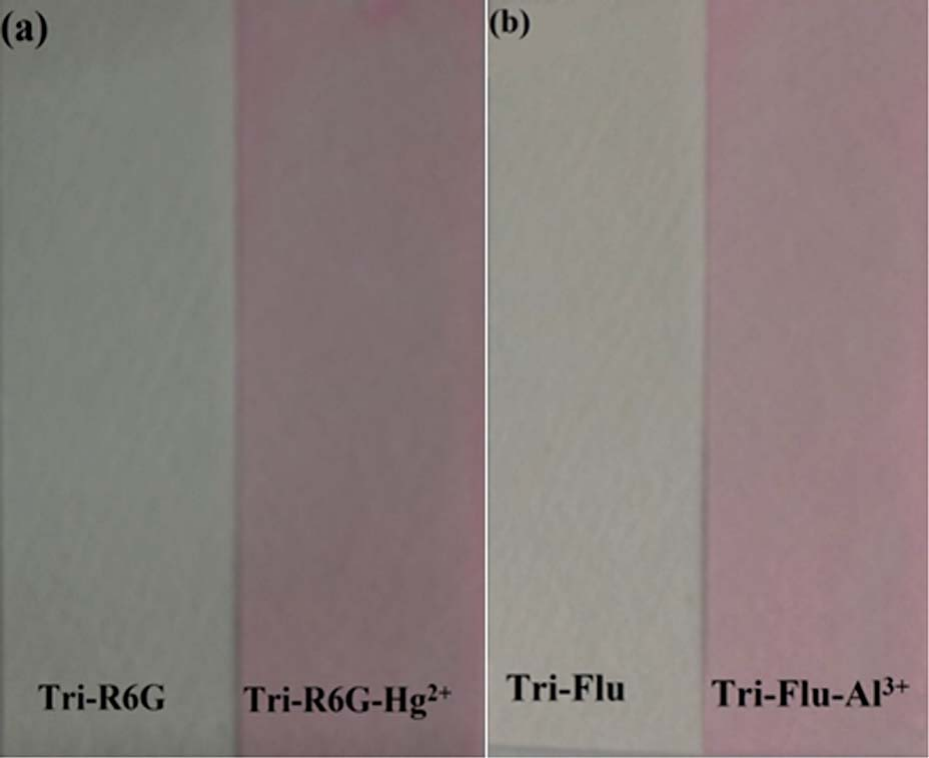
Photographs showing the color changes of sensor Tri-R6G (a) and Tri-Flu (b) before and after addition of Hg^2+^ and Al^3+^ under sunlight, respectively.

In order to study the practical application of probes Tri-R6G and Tri-Flu, we dipped the filter paper into a solution of Tri-R6G or Tri-Flu, and then dried in the air. Subsequently, the test strip was treated with Hg^2+^ or Al^3+^ solution (mM) which resulted in an obvious color change from colorless to light pink. Therefore, the test strips could be conveniently utilized for detecting Hg^2+^ and Al^3+^ in solutions (Fig. 9).

**Fig. 10:**
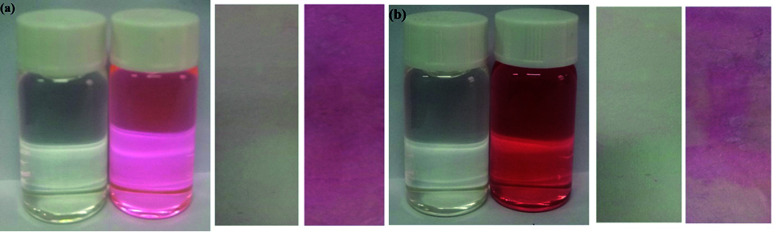
(a) Photographs showing the color change of the sensor Tri-R6G solution and test strips in a water sample containing no Hg^2+^ and water samples in the river. (b) Photographs showing the color change of the sensor Tri-Flu solution and test strips in a water sample containing no Al^3+^ and water samples in the river.

We compare the water in the river with the water without Hg^2+^. As shown in Fig. 10(a), the water in the river is added to the Tri-R6G probe solution. The color changes from colorless to pink, prepared by Tri-R6G. The test strip turns pink. Similarly, as shown in Fig. 10(b), probe Tri-Flu has same phenomenon withTri-R6G. These results demonstrate that the probe Tri-R6G has great promise to determine Hg^2+^ in a simple, convenient and portable way. Similarly, the same is true for probe Tri-Flu.

### 3.6 Binding sites

**Fig. 11:**
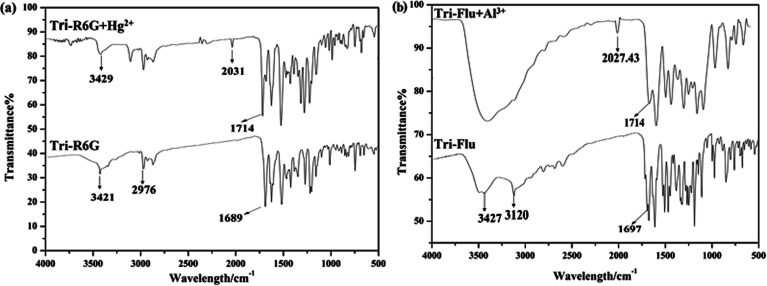
The FT-IR spectrum of Tri-R6G and Tri-R6G + Hg^2+^ (a); the FT-IR spectrum of Tri-Flu and Tri-Flu + Al^3+^ (b).

The FT-IR spectrum of probe Tri-R6G and complex Tri-R6G + Hg^2+^ were shown in Fig. 11(a). Free probe Tri-R6G showed stretching vibration bands assignable to N–H and C=N at 3429 and 1689 cm^−1^, respectively. While complexation of Tri-R6G with Hg^2+^ resulted in the obvious shift of N–H stretching vibration band from 3429 cm^−1^ to 3421 cm^−1^, and the tretching vibration band of C=N shifted to 1714 cm^−1^. Similarly, the IR spectrum of probe Tri-Flu and Tri-Flu + Al^3+^ were shown in Fig. 11(b). Free probe Tri-Flu showed stretching vibration bands assignable to O–H and C=N at 3427 and 1697 cm^−1^, respectively. When Al^3+^ is added, the stretching vibration band of C=N shifted to 1714 cm^−1^.

**Fig. 12:**
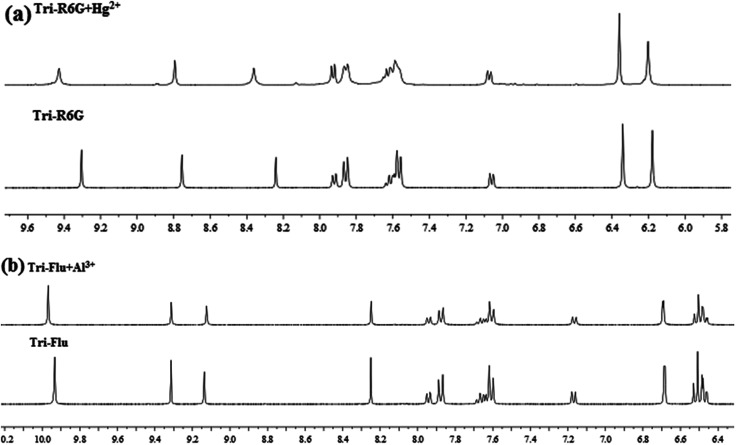
The ^1^H NMR of Tri-R6G and Tri-R6G + Hg^2+^ (a); the ^1^H NMR spectrum of Tri-Flu and Tri-Flu + Al^3+^ (b).

**Fig. 13:**
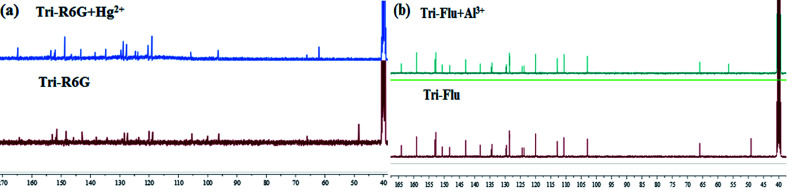
The ^13^C NMR of Tri-R6G and Tri-R6G + Hg^2+^ (a); the ^13^C NMR spectrum of Tri-Flu and Tri-Flu + Al^3+^ (b).

To further study the binding interactions of sensor Tri-R6G + Hg^2+^ and Tri-Flu + Al^3+^, ^1^H NMR titrations have been performed in DMSO-*d*_6_ as shown in Fig. 12. As shown in Fig. 12(a), upon addition of Hg^2+^ (1.0 μM) to the solution of sensor Tri-R6G, the H proton signals of C=N and –NH_2_ at 9.30, 8.75 and 8.24, respectively. But, after the addition of Hg^2+^ (1.0 μM) to the solution of sensor Tri-R6G, the H proton signals of C=N and –NH_2_ shifted to higher *δ* values (9.43, 8.79 and 8.36 ppm) with respect to sensor Tri-Flu. Under the same conditions, upon addition of Al^3+^ (1.0 μM) to the solution of sensor Tri-Flu, the H proton signal of phenolic O–H located and H proton signal of aldimine C=N shifted from *δ* 9.93 and 9.12 ppm to 9.96 and 9.14 ppm, respectively. To further study the binding interactions of sensor Tri-R6G + Hg^2+^ and Tri-Flu + Al^3+^, ^13^C NMR titrations have been performed in DMSO-*d*_6_ as shown in Fig. 13. In the Fig. 13(a), after the addition of Hg^2+^ (1.0 μM) to the solution of sensor Tri-R6G, the C proton signals of C=N from 62 ppm to 48 ppm. Similarly, the addition of Al^3+^ (1.0 μM) to the solution of sensor Tri-Flu, the C proton signals of C=N from 56 ppm to 47 ppm in the Fig. 13(b). These data strongly draw the conclusion that when the probe binds with metal ions, the complexation reaction occurs at C=N position, which leads to the migration of intramolecular electrons and the H proton signals of C=N, –OH and –NH_2_ generated offset, and the C proton signal of C=N is shifted.

**Scheme 2:**
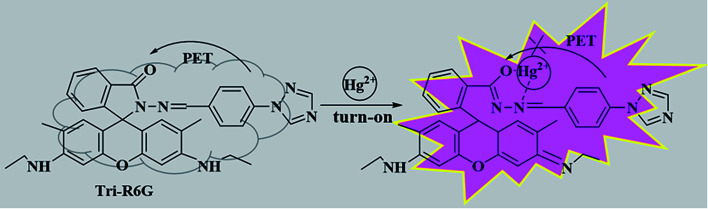
The proposed sensing mechanism of sensor Tri-R6G and Hg^2+^.

Based on Job's plot and FT-IR, we predict the possible binding mechanism of probes with metal ions as shown in Scheme 2.^55–57^ For free probe Tri-R6G, the photoelectron transfer (PET) process leads to the fluorescence quenching of rhodamine. Before the metal ions are bound, the sensor molecules appear to be fluorescently quenched. When the rhodamine hydrazide group is combined with the metal ion, the electron donating ability of the rhodamine hydrazide group is reduced, and the PET process is suppressed. The electrons excited by light in the fluorescent group can directly jump back to the original ground state orbital, thereby enhanced fluorescence emission of the fluorophore. Addition of Hg^2+^ causes the ring opening of lactam and the blocking of the PET process described above. Similarly, the complications of Al^3+^ with Tri-Flu blocks the PET process in fluorescein matrix, thereby causing obvious fluorescence enhancement.

### 3.7 Theoretical calculation

**Fig. 14:**
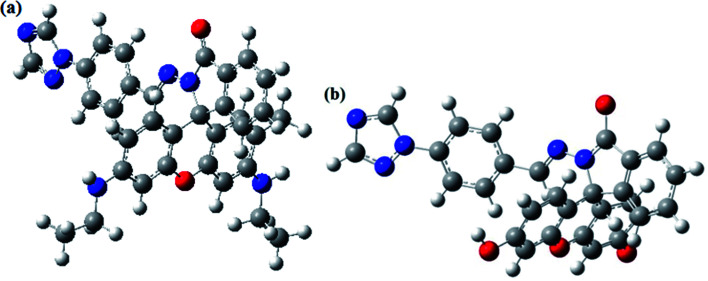
The optimized molecular structures of Tri-R6G (a) and Tri-Flu (b).

**Fig. 15:**
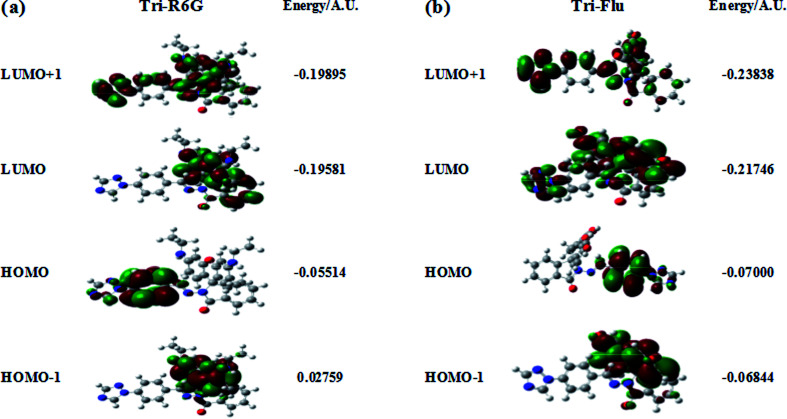
Corresponding orbital electron distribution of probes Tri-R6G (a) and Tri-Flu (b).

The optimized molecular structure, molecular orbital distribution and excitation energies of the synthetic probes were calculated by Gaussian 09 program and the density functional theory (DFT) at B3LYP/6-311++g(d.p) level. The optimized structures of probe Tri-R6G and Tri-Flu were shown in Fig. 14. The energy level, orbital distribution and orbital energy were listed in Fig. 15. The location of HOMO electron cloud of probe Tri-R6G is mainly at the benzene ring, as show in Fig. 15(a), theHOMO−1 and LUMO orbital electron clouds were populated on the Rhodamine 6G matrix, whereas the electron cloud of LUMO+1 almost delocalized over the whole structure. The overlap between them indicated that the electron transition could be easily implemented. The orbital energy of probe Tri-R6G increased in the order of HOMO−1 (0.02759), HOMO (−0.05514), LUMO (−0.19581) and LUMO+1 (−0.19895) were sequentially increased, also the energies of HOMO, LUMO, LUMO+1 were negative. It could be predicted that probe Tri-R6G could easily combined with metal cations. Probe Tri-Flu has a simpler electronic transition than probe Tri-R6G. In Fig. 15(b), the electron clouds of HOMO−1 and HOMO were delocalized on the fluorescein matrix, and the electron clouds of LUMO and LOMO+1 were mainly located the entire structure. Similarly, the orbital energy of probe Tri-Flu increased in the order of HOMO−1 (−0.06844), HOMO (−0.07000), LUMO (−0.21746), LUMO+1 (−0.23838) were sequentially increased and negative. It was predicted that probe Tri-Flu might readily bind to metal cations. We also calculated the orbital energy differential of probe Tri-R6G and Tri-Flu were small, the results are shown in Table 1, which means that the electrons can be easily transitioned.

**Table 1:** Orbital energy differential of Tri-R6G and Tri-Flu

Compound	Δ*E*_H → L_(AU)	*E*_H → L+1_(AU)	*E*_H−1 → L_(AU)
Tri-R6G	0.14067	0.14381	0.16822
Tri-Flu	0.14746	0.16838	0.14902

## 4. Conclusion

In summary, two kinds of novel fluorescent probes were synthesized and proved to have high applicability, low detection limit and easy-to-handle. The properties of the probes were predicted by theoretical calculation, and their UV-vis and fluorescence properties were also investigated and the results demonstrated that the probes have specificity for Hg^2+^ and Al^3+^ over other detected metal ions based on the PET mechanism, respectively. In addition, these two probes are extremely resistant to interference from other metal ions, and can visually detect metal ions from white to pink in solution or test paper.

## Conflicts of interest

There are no conflicts to declare.

## Supplementary Material

RA-010-C9RA08428B-s001
